# Multifarious topological quantum phase transitions in two-dimensional topological superconductors

**DOI:** 10.1038/srep28471

**Published:** 2016-06-22

**Authors:** Xiao-Ping Liu, Yuan Zhou, Yi-Fei Wang, Chang-De Gong

**Affiliations:** 1 National Laboratory of Solid State Microstructures and Department of Physics, Nanjing University, Nanjing, 210093, China; 2 Condensed Matter Physics and Material Science Department, Brookhaven National Loboratory, Upton, New York 11973, USA; 3 Center for Statistical and Theoretical Condensed Matter Physics and Department of Physics, Zhejiang Normal University, Jinhua 321004, China

## Abstract

We study the two-dimensional topological superconductors of spinless fermions in a checkerboard-lattice Chern-insulator model. With the short-range *p*-wave superconducting pairing, multifarious topological quantum phase transitions have been found and several phases with high Chern numbers have been observed. We have established a rich phase diagram for these topological superconducting states. A finite-size checkerboard-lattice cylinder with a harmonic trap potential has been further investigated. Based upon the self-consistent numerical calculations of the Bogoliubov-de Gennes equations, various phase transitions have also been identified at different regions of the system. Multiple pairs of Majorana fermions are found to be well-separated and localized at the phase boundaries between the phases characterized by different Chern numbers.

Topological superconductors (TSCs) have been a hot topic in recent a few years due to their unique properties and potential applications^1^. One of the most significant characteristics of TSCs is Majorana fermions (MFs) which are found to exist in vortices^2^,^3^,^4^,^5^ or on the boundaries^6^,^7^,^8^,^9^,^10^,^11^,^12^,^13^,^14^,^15^ of the TSC system. MFs are their own anti-particles^16^,^17^. They obey non-Abelian statistics^4^ and could be used in topological quantum computation^18^,^19^, which is also a significant reason for the recent wide concern on TSCs.

Generally, two proposals are suggested to realize TSCs and MFs, either with the spin-triplet *p*-wave superconductivity^6^,^7^,^20^,^21^,^22^ or with the conventional *s*-wave superconductivity by proximity effect in some materials^8^,^9^,^10^,^11^,^12^,^13^,^14^,^15^,^23^,^24^,^25^,^26^,^27^,^28^,^29^,^30^,^31^,^32^. The simplest and most straightforward model systems are the one-dimensional spinless *p*-wave TSCs or two-dimensional *p*_*x*_ ± *ip*_*y*_ TSCs. For example, Read and Green^20^ considered a chiral TSC state with a Chern number^33^ 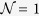 for *p*_*x*_ ± *ip*_*y*_ paired spinless fermions. In a one-dimensional quantum wire model by Kitaev^6^, MFs were found to locate at the ends of the chain. Possible topological quantum phase transitions (TQPTs) among TSCs and conventional superconducting or insulating states are also very intriguing, and it is possible to identify MFs at the phase boundaries of TQPTs. However, previous works^6^,^7^,^20^,^21^,^22^ are mostly done based upon models with topological pairing of fermions in a single topologically trivial band and varieties of TSC phases seem more or less limited, and additional condition such as long-range superconducting pairing^21^,^22^ is required in some cases to achieve other TSC phases. Recently, Qi *et al.* proposed that when a quantum Hall (QH) state or a quantum anomalous Hall (QAH) state near the plateau transition of *N* to *N* − 1 is coupled to a conventional *s*-wave superconductor through the proximity effect, a new TSC phase with a Chern number 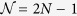 will appear between the phase 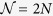 and 2*N* − 2. Their work illustrates that a nontrivial TSC phase with a Chern number 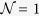 or 2 is achievable through TQPTs from trivial phases^30^.

In this paper, we address a two-dimensional TSC model of paired spinless fermions (or spin-polarized electrons) on a Chern insulator (CI) with QAH states and focus on the TQPTs that will possibly occur. The checkerboard-lattice CI/QAH model which has two nontrivial topological energy bands is our preferred candidate system. We only consider the short-range nearest-neighbor superconducting pairing. The possible phase diagram has been explored, and rich and interesting TQPTs have been identified. Various TSC phases with the Chern numbers ranging from 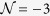 to 3 are found. Then, we further investigate the signatures of TQPTs for this checkerboard-lattice model on a finite-size cylinder. A harmonic potential trap which can be easily manipulated in cold atom systems is imposed to regulate the fermion density at lattice sites. According to the numerical results obtained from solving the Bogoliubov-de Gennes (BdG) equations self-consistently, TQPTs are also clearly identified; MFs are found to be spatially well-separated and distributed near the phase boundaries inside the system; the zero-energy local density of states (LDOS) is observed to exhibit prominent peaks on the phase boundaries which is suggested to be a signature for observing MFs in experiments.

## Results

### The lattice model

**Figure 1:**
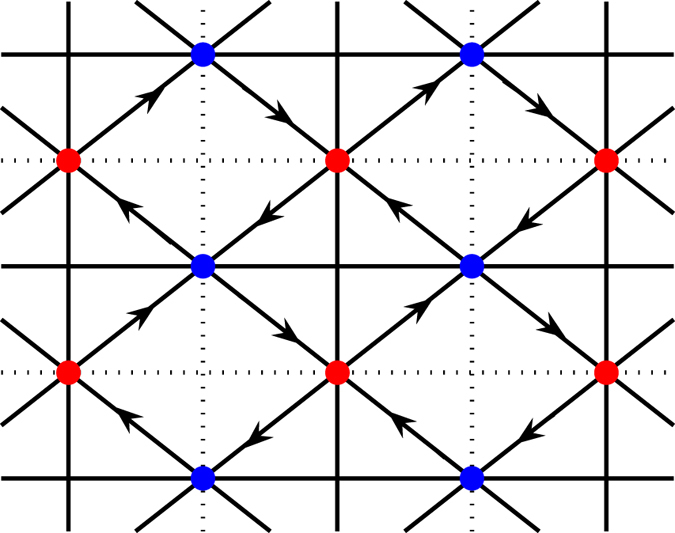
The checkerboard-lattice CI/QAH model. Sites in red and blue constitute two sublattices respectively. Staggered fluxes are superimposed on the plaquettes, resulting in an additional phase factor ±*ϕ* on the nearest-neighbor hopping (± denoted by the arrow direction). Solid and dotted lines, connecting the second-nearest-neighbor sites (same sublattice), denote the hopping parameters 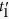 and 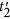.

The minimal CI/QAH model in the checkerboard lattice with nontrivial topological bands^34^,^35^ we adopt is illustrated in Fig. 1. We consider *p*_*x*_ + *ip*_*y*_ pairing between spinless fermions in the nearest-neighbor pairs of sites. The concerned Hamiltonian is therefore written as


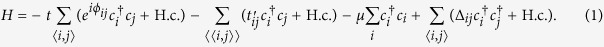


Here, *c*_*i*_ 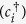 annihilates (creates) a spinless fermion on the site *i*. Staggered fluxes are superimposed in the plaquettes and induce additional phase factors ±*ϕ* on the nearest-neighbor hopping *t*. This means the *C*_4_ rotational symmetry is broken, leading to two sublattices *A* (red) and *B* (blue). The second-nearest-neighbor hopping takes different value 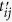 (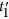, and 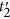 for solid, and dotted connection, respectively). *μ* is the chemical potential. 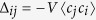 is the pairing potential with *V* the strength of the attractive interaction. We set *t* = 1 as unit and 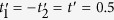 in the calculations. In fact, our main results are not sensitive to the selected parameters.

In the normal state, the system is a CI/QAH state with two topological bands of Chern numbers *N* = ±1 when *ϕ* ≠ *nπ*^35^. Particularly, one of the two bands can be tuned to be very flat with the third-nearest-neighbor hopping, which hosts new fractional quantum Hall states^36^,^37^.

### TQPTs and phase diagram of TSCs

In order to explore the possible TQPTs, we solve Eq. (2) (See Methods) with different parameters at fixed *p*_*x*_ + *ip*_*y*_ pairing order parameter Δ = 0.1. The Hamiltonian can be diagonalized under a rational transformation and further a Bogoliubov transformation. In principle, the distinct TSC phases can be characterized by the Chern numbers of two lower BdG bands (*C*_1_, *C*_2_) (also two higher BdG bands with *C*_3_ = −*C*_2_ and *C*_4_ = −*C*_1_ due to particle-hole symmetry). However, the individual Chern number is not well defined in some cases (especially for small |*μ*|) due to overlap of the two lower BdG bands. We use the sum of the two Chern numbers 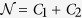 to characterize the TSC phases.

The Chern number of each BdG band can be calculated with those BdG wave functions numerically^33^. A sophisticated way is to find out the condition under which the two middle BdG bands touch and re-open. The phase boundaries are *μ* = ±4 sin *ϕ* with a single Dirac point (*π*, *π*), *μ* = ±4 cos *ϕ* with a single Dirac point (0, 0), and 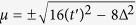 with two Dirac points (*π*, 0) and (0, *π*). Numerical calculations show that each Dirac point carries a Berry phase ±*π* and the total Berry phase is ±2*π* for the case of two symmetric Dirac points with the same sign Berry phases. Therefore, when the system crosses a phase boundary, a TQPT occurs with the changed Chern number 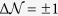, or ±2 for single, and double Dirac points, respectively.

**Figure 2:**
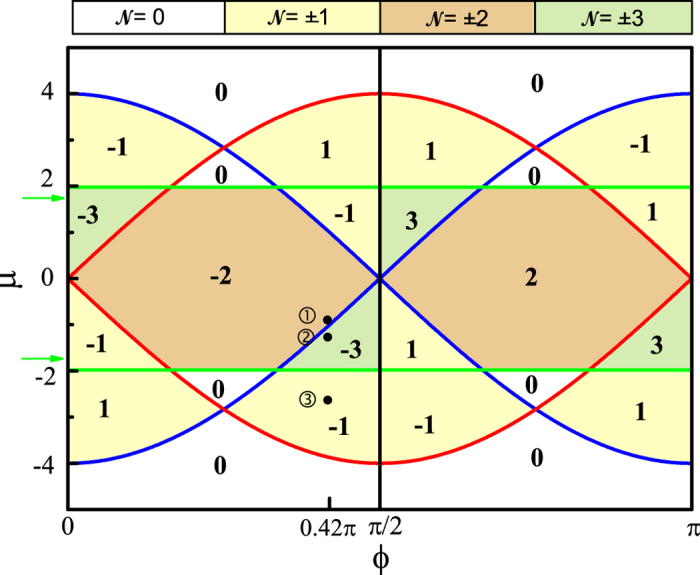
Phase diagram in the (*ϕ*, *μ*) parameter space. The pairing amplitude is fixed to Δ = 0.1. Red, blue, and green lines represent three phase boundaries with *μ* = ±4 sin *ϕ*, *μ* = ±4 cos *ϕ*, and 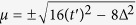, respectively. The TSC Chern numbers 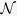 are specified. Green arrows mark the slightly shifted positions of the phase boundaries in green for a fixed Δ = 0.35. Black dots with serial numbers will be discussed in the next subsection.

The overall phase diagram of TSCs with the characteristic Chern numbers 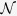 ranging from −3 to 3 is depicted in Fig. 2, showing multifarious TQPTs. In fact, for small enough *μ* (uncolored region), the system is the same as the single band *p* + *ip* superconductor with strong pairing suggested by Read and Green^20^ irrespective of the topology of the two normal-state bands. Here, the high Chern number TSC is naturally produced without applying additional condition, for example the long-range superconducting pairing^21^,^22^. The topological phase transition in present CI with 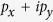 superconductivity is much richer than that in the QH/QAH states coupled to a conventional *s*-wave superconductor through the proximity effect^30^,^31^. Besides the previous suggested 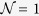 TSC phase between the 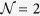 TSC phase and 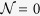 superconducting phase, TSC phases with 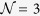 (or −3) also emerge via a series of TQPTs.

Phase diagrams with 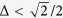 are almost the same except that the phase boundaries of 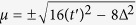 (green lines in Fig. 2) have slight shifts. These two phase boundaries disappear when 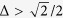, resulting in the limited TQPTs with Chern numbers 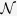 ranging from −1 to 1. We will not illustrate these cases in detail here. We also remark that the corresponding phase diagram for *p*_*x*_ − *ip*_*y*_ pairing with Δ = 0.1 is almost the same as Fig. 2 except that the Chern numbers of the positive and negative *μ* region exchange with each other.

### Cylinder with a harmonic trap

To better understand the TQPTs that occur in our model, and more importantly, the nature of induced MFs, which are proposed to be generated on the phase boundaries of this quasi-one-dimensional system^7^,^38^, we study the present model in real space. A harmonic potential trap^39^ 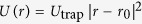 (here *r* − *r*_0_ has a unit length 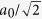 with *a*_0_ = 1 the length of a unit cell) is imposed on a finite-size *L*_*x*_ × *L*_*y*_ = 400 × 8 checkerboard lattice, which introduces an effective chemical potential 
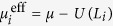
 at a site *i*. The fermion density can be regulated and various TQPTs as well as phase boundaries in Fig. 2 can be induced by varying the trap potential.

Both the *x* and *y* lattice directions are treated with periodic boundary conditions. Due to the puny size of the lattice in *y* direction compared with the large size in *x* direction, in the numerical calculations, we will neglect the *y* dependence and only consider the *x* dependence of physical quantities. The trap center is located at *L*_0_ = 200. By solving the BdG equations self-consistently, some interesting results are revealed below.

**Figure 3:**
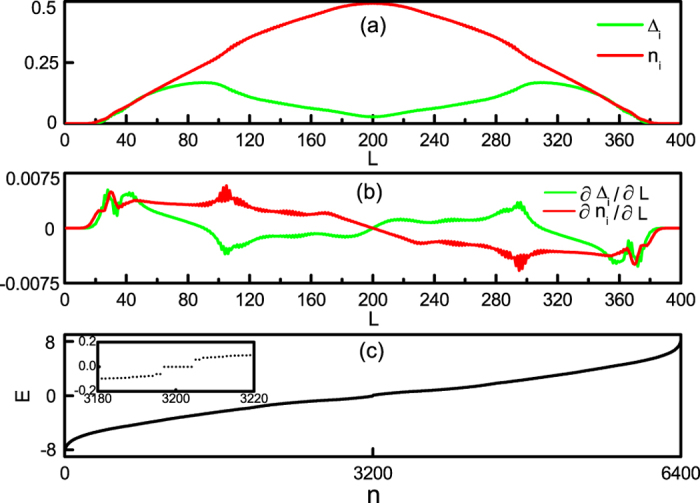
Cylinder system. (**a**) Superconducting order parameter (green line) and fermion density (red line) along lattice sites. (**b**) First-order derivatives of order parameter (green line) and fermion density (red line) versus sites. (**c**) Energy spectrum of the lattice. Low-energy spectrum is magnified in the inset. Parameters are *V* = 1.75, *ϕ* = 0.42*π*, *μ* = −0.9, *U*_trap_ = 0.00018.

We select the parameters *ϕ* = 0.42*π*, *V* = 1.75, *μ* = −0.9, *U*_trap_ = 0.00018 to study the TQPTs and the corresponding MFs. The fermion density *n*_*i*_ [red line in Fig. 3(a)] decreases monotonically to zero from the center to the left/right edge, which is well confined by the trap potential. In comparison, the superconducting order parameter Δ (green line) exhibits interesting site dependence. Its amplitude increases from a relative smaller value at the center site (*L*_*i*_ = 200), and reaches a maximum value at a critical length (*L*_*i*_ = 90, 310). The superconducting gap then weakens down to zero upon further moving outwards. The non-monotonic behavior can be understood as follows. The energy scales of the two normal-state bands are ascertained to 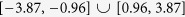 at *ϕ* = 0.42*π*. The on-site potential *U*(*L*_*i*_) is weak enough for 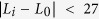 and the corresponding effective chemical potential 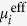 stays in the normal-state band gap. Small density of states near Fermi energy naturally leads to weak superconducting order parameters. The density of states increases when 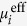 crosses the lower normal-state band with increasing |*L*_*i*_ − *L*_0_|. However, 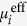 locates below the lower band for 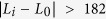, resulting in the near zero value of Δ. Here, we emphasize that the superconducting gap Δ near the edge in the *p* + *ip* TSC is always zero regardless whether the trap potential is applied due to lack of counterpart edge state. This differs from the on-site *s*-wave pairing in HgTe quantum well^40^ and QAH state with a conventional *s*-wave superconductivity proximity effect^30^, where the strongest superconductivity locates near the edge.

In fact, the possible TSC phase transitions are well illustrated within the present trap potential. From the center to the left/right edge, the system undergoes the TSC phases with 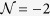, −3, −1 respectively, and then the insulating phase 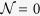 with decreasing 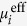, consisting with the notations in Fig. 2. This process can also be signified by the first-order derivatives of the superconducting order parameter and the fermion density [Fig. 3(b)]. A few peaks can be identified, corresponding to the above mentioned TSC phase transitions.

**Figure 4:**
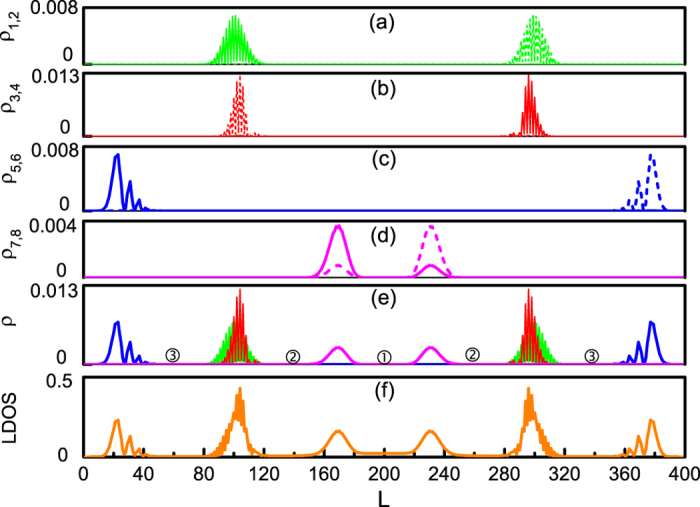
MFs in cylinder system. (**a**–**d**) Display distributions of the corresponding MFs. Solid and dash lines are used to distinguish MFs in the same pair. (**e**) Summary of distributions of all the MFs. Here, |*ρ*_*i*_ − *ρ*_*i*+1_| (*i* = 1, 3, 5, 7) is adopted to eliminate the overlapping. Equivalent positions of sites ➀ (*L* = 200), ➁ (*L* = 140, 260), ➂ (*L* = 60, 340) are marked in Fig. 2. (**f**) Zero-energy LDOS along *x*-direction.

**Figure 5:**
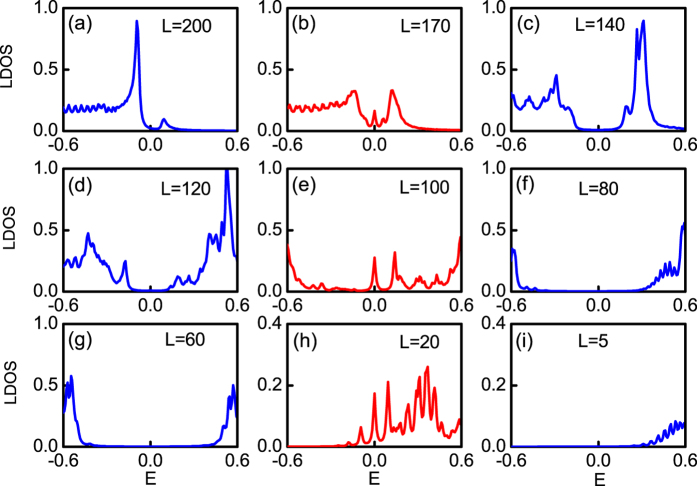
LDOS at different sites of cylinder system. (**a**) *L* = 200, (**b**) *L* = 170, (**c**) *L* = 140, (**d**) *L* = 120, (**e**) *L* = 100, (**f**) *L* = 80, (**g**) *L* = 60, (**h**) *L* = 20, (**i**) *L* = 5. LDOS at sites [(**b**), (**e**), (**h**)] lying on the phase boundaries are plotted by red lines.

In general, the MFs emerge near the phase boundary between the different TSC or TSC and insulating phases, as well as near the edge of the TSC phases. In Fig. 3(c), we output all 6400 eigenvalues of this cylinder system. The middle energy spectrum and four pairs of zero-energy modes protected by an energy gap about 0.06 can be clearly observed in the insert. As we know, for the *E* = 0, one fermion can be separated into two MFs which are expressed as 
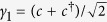
 and 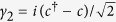 with *c*^†^ the zero-energy fermion^16^,^17^. According to zero modes, we plot spatial distributions of the corresponding four pairs of MFs in Fig. 4. Two MFs of each pair are well-separated and locally distributed on the lattice. Distributions of all the MFs are summarized in Fig. 4(e) where a subtraction |*ρ*_*i*_ − *ρ*_*i*+1_| (*i* = 1, 3, 5, 7) is adopted to eliminate the overlapping between the MFs in the same pair. The locations of the MF peaks in Fig. 4(e) are the same as those of the peaks in Fig. 3(b), further manifesting the TQPTs in TSC and MFs emerging near the phase boundaries. Moreover, the effective chemical potential where the MF peaks locate also corresponds to the phase boundaries *μ* = −4 cos *ϕ*, 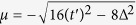 and *μ* = −4 sin *ϕ* as we discussed in the above section. The TQPTs and MFs can also be verified by the zero-energy LDOS^11^ along the lattice sites, where six peaks exist with the positions same as those of the MFs, and peaks of the first-order derivative of the order parameter. We further show the LDOS at some selected sites in Fig. 5. A zero energy mode peaked at *E* = 0 emerges at the sites near phase boundaries [(b), (e), (h)], but is absent in other sites, supporting our analysis.

## Summary and Discussion

In conclusion, we illustrate the multifarious TQPTs of two-dimensional spinless fermions with *p*-wave superconducting pairing on a checkerboard-lattice CI/QAH model. Various TSC phases, especially with high Chern numbers, are established with just short-range nearest-neighbor pairing. A rich TSC phase diagram is revealed by tuning the chemical potential and staggered-flux phase factor. This is in sharp contrast to the previous theoretical works based on single-band *p* + *ip* TSC or QAH states coupling to a conventional *s*-wave superconductor through the proximity effect. Furthermore, with the recent development on generating artificial gauge fields^41^ and especially realizing artificial staggered fluxes in optical lattices^42^,^43^,^44^,^45^, such a checkerboard-lattice CI/QAH model could be potentially realized in the near future. A finite-size checkerboard-lattice cylinder with a confining harmonic potential trap has been further explored using the self-consistent BdG method. Well-separated multiple pairs of MFs emerge at topological phase boundaries, identifying the TQPTs. These MFs are well controllable and robust against perturbations, and might be used for implementing the non-Abelian statistics and topological quantum computation^18^,^19^. In addition, such a checkerboard-lattice model might carry a topological flat band at selected model parameters which is beneficial to the superconducting pairing due to the large density of states. Future work might be very interesting to explore the competition of these TSC phases with other possible phases involved with the topological flat band.

## Methods

For the system with the periodic boundary conditions, the Hamiltonian can be rewritten in momentum space as


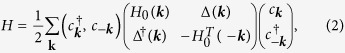


where 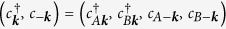. 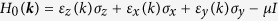 with 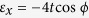

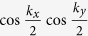
, 
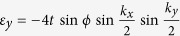
, and 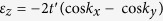. *σ* and *I* are the Pauli matrix and unit matrix, respectively. The *p*_*x*_ + *ip*_*y*_ pairing in momentum space is 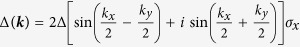 with Δ the superconducting order parameter.

When the lattice system has a finite size *L*_*x*_ × *L*_*y*_, we solve the BdG Hamiltonian self-consistently in real space


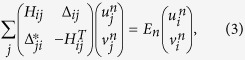


where 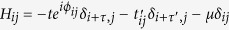 is the single particle Hamiltonian with *τ* and *τ*′ vectors linked to the nearest-neighbor and second-nearest neighbor sites. 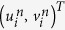 is the quasiparticle wave function corresponding to the eigenvalue *E*_*n*_. Due to the particle-hole symmetry of the BdG equations, the wave vector 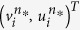 is also an eigenvector corresponding to eigenvalue −*E*_*n*_. The superconducting order parameter, on-site particle number *n*_*i*_, and the LDOS *ρ*_*i*_(*ω*), are determined self-consistently by


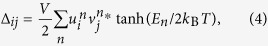



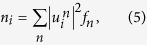



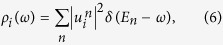


respectively with *f*_*n*_ as the Fermi distribution function.

## Additional Information

**How to cite this article**: Liu, X.-P. *et al.* Multifarious topological quantum phase transitions in two-dimensional topological superconductors. *Sci. Rep.* **6**, 28471; doi: 10.1038/srep28471 (2016).
